# Treatment Algorithm in Cancer-Associated Thrombosis: Updated Canadian Expert Consensus

**DOI:** 10.3390/curroncol28060453

**Published:** 2021-12-18

**Authors:** Marc Carrier, Normand Blais, Mark Crowther, Petr Kavan, Grégoire Le Gal, Otto Moodley, Sudeep Shivakumar, Deepa Suryanarayan, Vicky Tagalakis, Cynthia Wu, Agnes Y. Y. Lee

**Affiliations:** 1 Department of Medicine, Ottawa Hospital Research Institute, University of Ottawa, Ottawa, ON K1H 8L6, Canada; glegal@toh.ca; 2 Department of Medicine, Centre Hospitalier de l’Université de Montréal, Montreal, QC H2L 4M1, Canada; normand.blais.med@ssss.gouv.qc.ca; 3 Department of Medicine, McMaster University, Hamilton, ON L8N 4A6, Canada; crowthrm@mcmaster.ca; 4 Department of Oncology, Jewish General Hospital, McGill University, Montreal, QC H3T 1E2, Canada; petr.kavan@mcgill.ca; 5 Department of Hematology, Royal University Hospital, Saskatoon, SK S7N 0W8, Canada; otto.moodley@saskcancer.ca; 6 Department of Medicine, QEII Health Sciences Centre, Dalhousie University, Halifax, NS B3H 3A7, Canada; sudeep.shivakumar@nshealth.ca; 7 Department of Medicine, University of Calgary, Foothills Hospital, Calgary, AB T2N 2T9, Canada; deepa.suryanarayan@albertahealthservices.ca; 8 Department of Medicine, Centre for Clinical Epidemiology, Lady Davis Institute for Medical Research, Montreal, QC H3T 1E2, Canada; vicky.tagalakis@mcgill.ca; 9 Department of Medicine, University of Alberta, Edmonton, AB T5J 2J7, Canada; cwu@ualberta.ca; 10 Department of Medicine, University of British Columbia, British Columbia Cancer Agency, Vancouver, BC V5Z 4E6, Canada; alee14@bccancer.bc.ca

**Keywords:** cancer-associated thrombosis, venous thromboembolism, pulmonary embolism, anticoagulation

## Abstract

Patients with cancer-associated thrombosis (CAT) are at high risk of recurrent venous thromboembolism (VTE) and major bleeding complications. Risks vary significantly between individuals based on cancer status, treatment, and other characteristics. To facilitate the evidence-based management of anticoagulant therapy in this patient population, a committee of 11 Canadian clinical experts updated a consensus-based algorithm for the acute and extended treatment of symptomatic and incidental CAT that was developed in 2018. Following a systematic review of the literature, updates to the algorithm were discussed during an online teleconference, and the algorithm was subsequently refined based on feedback from committee members. Clinicians using this treatment algorithm should consider bleeding risk, type of cancer, and drug–drug interactions, as well as patient and clinician preferences, in tailoring anticoagulation for patients with CAT. Anticoagulant therapy should be adapted as the patient’s cancer status and management change over time.

## 1. Introduction

The management of venous thromboembolism (VTE) is a frequent and important clinical issue in patients with cancer. The 6-month VTE risk for patients with cancer is 12-fold higher compared to the general population, and as much as 23-fold higher in patients receiving chemotherapy or targeted therapy [1]. Over the past two decades, the 12-month cumulative incidence for VTE has increased three-fold in cancer patients [1]. Furthermore, thromboembolism has been reported to be the second leading cause of death in patients with cancer, highlighting the importance of urgently initiating therapeutic dosing of anticoagulation [2,3]. However, the management of anticoagulant therapy for cancer-associated thrombosis (CAT) is complex due to an increased risk of both recurrent VTE and major bleeding in patients with cancer as compared to those without cancer [4,5]. Selection and dosing of anticoagulant therapy for CAT needs to be individualized based on the patient’s risk for both recurrent VTE and bleeding. This can be influenced by patient characteristics, type and stage of cancer, and anticancer treatment [4,5].

Several classes of anticoagulants have been studied in the treatment of CAT, including vitamin K antagonists (VKAs), subcutaneous low-molecular-weight heparins (LMWHs), and direct oral anticoagulants (DOACs). For many years, LMWHs were the standard of care for the acute and extended treatment of CAT based on the results of trials comparing LMWH with VKA [6,7,8,9]. Patients with cancer were underrepresented in initial trials comparing DOACs to VKA for the acute treatment of VTE [10,11,12,13,14,15]. However, the recent publication of clinical trials comparing DOACs with LMWH in the cancer-patient population has expanded the therapeutic options for the acute and extended treatment of CAT, but also introduced a layer of complexity [16,17,18]. To provide guidance to health care professionals on tailoring anticoagulant treatment in patients with CAT, an evidence-based risk stratification treatment algorithm was developed in 2018 [19]. The consensus process reported here was undertaken to up-date this treatment algorithm based on the evolving body of evidence.

## 2. Methods

### 2.1. Literature Review

To update the treatment algorithm, the committee used, as a starting point, the previously published 2018 Canadian expert consensus treatment algorithm in CAT [19]. A systematic review of the literature published since 2018 was performed using the search strategies outlined in supplemental Appendix A. All abstracts were reviewed. References of narrative reviews identified by the search strategy also were reviewed to ensure that all potentially relevant articles were captured.

### 2.2. Revision of Treatment Algorithm

The multidisciplinary consensus group, which included 11 physicians with expertise in the areas of hematology, medical oncology, and general internal medicine, met in April 2021 via Web-based teleconference to discuss how the results of the literature search would impact the 2018 treatment algorithm. The revised treatment algorithm and manuscript were then circulated by email on two occasions so that committee members could review them and provide comments. The final version of updated algorithm was approved by all members.

### 2.3. Role of the Funding Sources

The revision of the algorithm was funded by unrestricted grants from Pfizer Canada, Hospital Business Unit (Kirkland, QC, Canada), Bayer Canada Inc (Montreal, QC, Canada), LEO Pharma Inc (Thornhill, ON, Canada) and Servier Canada (Ottawa, ON, Canada) to Thrombosis Canada. The authors administered all aspects of revising the treatment algorithm, and the funding sources had no role in drafting, editing, or approving the treatment algorithm.

## 3. Results

**Figure 1:**
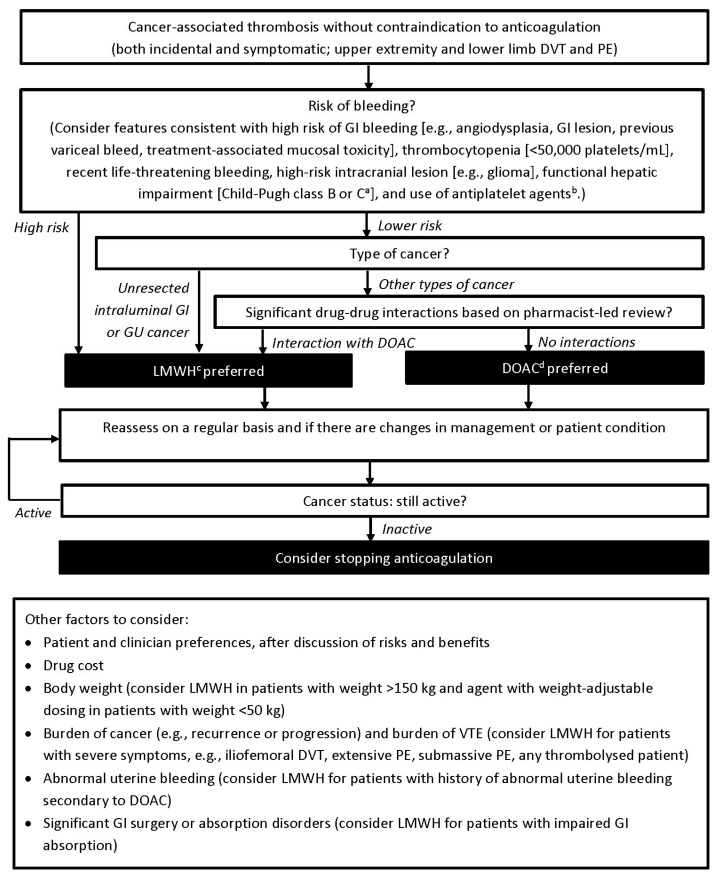
Patient risk stratification algorithm for anticoagulant therapy in cancer-associated thrombosis. ^a^ None of the DOACs are recommended for use in patients meeting criteria for Child-Pugh class C, with use of rivaroxaban being contraindicated in patients with hepatic disease (including Child-Pugh class B and C) associated with coagulopathy and having clinically relevant bleeding risk. Apixaban should be used with caution in patients with mild or moderate hepatic impairment (Child-Pugh class A or B), while these patients exhibited comparable pharmacokinetics and pharmacodynamics to healthy controls when treated with edoxaban. ^b^ Use of antiplatelet agents should be assessed, and discontinuation should be considered in the absence of a strong indication. Shared decision-making with other health care providers is warranted. ^c^ Currently, dalteparin, enoxaparin, and tinzaparin have randomized controlled trial evidence in cancer-associated thrombosis, with the evidence base being stronger for dalteparin and tinzaparin. Refer to the relevant product monograph for appropriate dosing. ^d^ Currently, apixaban, edoxaban, and rivaroxaban have randomized controlled trial evidence in cancer-associated thrombosis, with stronger evidence for apixaban and edoxaban. Refer to the relevant product monograph for appropriate dosing. DVT = deep vein thrombosis; PE = pulmonary embolism; GI = gastrointestinal; GU = genitourinary; DOAC = direct-acting oral anticoagulant; LMWH = low molecular weight heparin; VTE = venous thromboembolism.

The search of the PubMed database identified 420 articles, 22 of which were selected for review according to pre-specified criteria, while the search of the American Society of Hematology abstract database identified 27 abstracts, one of which was selected for review. The committee included an additional 12 articles not identified by the search strategy. Following review of the 35 selected articles and abstracts and discussion of the new evidence, the revisions to the algorithm were recommended. The resulting treatment algorithm (Figure 1) provides guidance on the selection of anticoagulant therapy for cancer patients with incidental or symptomatic upper extremity or lower limb deep vein thrombosis (DVT) or pulmonary embolism (PE).

According to the treatment algorithm, LMWH is preferred in patients at high risk of bleeding, with unresected intraluminal gastrointestinal (GI) or genitourinary (GU) cancer, or with significant drug-drug interactions with DOACs. In contrast, DOACs are preferred in patients at low risk of bleeding, with other cancer types, and without significant drug–drug interactions. Other factors to consider include patient and clinician preference, drug cost and coverage, body weight, burden of cancer, burden of VTE, and history of abnormal uterine bleeding, significant GI surgery, or absorption disorders. Anticoagulant therapy should be reassessed regularly (e.g., every 3 months), and sooner if there are changes in the patient’s management or condition. In general, anticoagulation should be continued in patients with active cancer (underlying cancer present or on-going anti-cancer treatment), while discontinuation may be considered in those whose cancer is no longer active.

## 4. Discussion

### 4.1. Efficacy and Safety of Anticoagulants

Until the publication of randomized controlled trials (RCTs) comparing DOACs with LMWH for the acute treatment of CAT (Table 1), clinical practice guidelines recommended the use of LMWH over DOACs or VKA for the acute and secondary prevention of VTE in patients with cancer [6,7]. Recent guidelines, on the other hand, have suggested that either a DOAC or LMWH can be used for the acute treatment of CAT [19,20,21,22,23], with recommendations that treatment be individualized based on patient characteristics.

A meta-analysis of the results of all RCTs comparing LMWH with VKA for the management of CAT reported a 44% reduction in the risk of recurrent VTE (relative risk (RR): 0.56; 95% confidence interval (CI): 0.43 to 0.74), without a significant increase in the risk of major bleeding (RR: 1.07; 95% CI: 0.66 to 1.79) in patients treated with LMWH [29]. A similar meta-analysis of the results of all RCTs comparing DOACs with LMWH for the treatment of acute CAT reported a significantly lower risk of recurrent VTE (hazard ratio (HR): 0.63; 95% CI: 0.47 to 0.86) and a non-significantly higher risk of major bleeding (HR: 1.26; 95% CI: 0.84 to 1.90) with DOACs as compared to LMWH [28]. An analysis of 29 studies including a total of 8000 patients with cancer found that case fatality rates were higher for recurrent VTE than those for major bleeding at 15.0% (95% CI 6.6 to 30.1%) and 8.9% (95% CI 3.5 to 21.1%), respectively [30]. Although case fatality rates varied by type of anticoagulation in this analysis, the differences were not statistically significant [30]. Taken together, these data highlight the importance of preventing recurrent VTE while minimizing the risk of major bleeding complications in patients with cancer. LMWH is more effective than VKA without any increase in bleeding complications. DOACs are non-inferior to LMWHs in terms of overall safety and efficacy. No data comparing the direct thrombin inhibitor dabigatran with LMWH for the management of CAT are available.

### 4.2. Incidental VTE

Although patients with incidental VTE (i.e., asymptomatic thrombosis found on screening imaging tests) were not included in the RCTs of LMWH vs. VKA [8,9,24,25,26], between 20% and 53% of patients included in the RCTs of DOACs vs. LMWH had incidental VTE at baseline [16,17,18]. Although the rate of recurrent VTE is lower in patients with incidental VTE compared to those with symptomatic events (RR: 0.62; 95% CI 0.44 to 0.87), the rate of recurrent events despite anticoagulation remains high [31]. For example, the results of a subanalysis of patients with incidental vs. symptomatic VTE in the Hokusai-VTE Cancer trial found recurrent VTE occurred in 7.9% of patients with incidental VTE as compared to 10.9% of those with symptomatic VTE [32]. Additionally, an analysis of data from the Swiss Venous Thromboembolism Registry (SWIVTER) reported that the rates of both mortality and VTE recurrence in these patients were lower if they received anticoagulation therapy for at least 3 months [33]. In this study, mortality rates were 4% in patients receiving anticoagulation as compared to 41% in those without anticoagulation, while recurrence rates were 1% and 18%, respectively [33]. These findings support managing patients with incidentally detected CAT in a similar manner as symptomatic CAT.

### 4.3. Upper Extremity and Catheter-Related VTE

Even though catheter-related VTE is a common complication in patients with cancer, there is limited evidence to guide the management of upper extremity and catheter-related VTE as these patients were excluded from all RCTs of LMWH vs. VKA and DOAC vs. LMWH except for the ADAM-VTE trial [8,9,16,17,18,24,25,26,27,28]. Two studies of cancer patients with upper extremity catheter-related DVT suggested that LMWH and VKA are safe and effective, with no recurrent VTE events reported in either study and major bleeding event rates of 4% and 2% at 3 months [34,35]. In contrast, a prospective cohort study evaluating rivaroxaban monotherapy in 70 cancer patients with upper extremity catheter-related DVT demonstrated a VTE recurrence rate of 1.4%, including one fatal PE, and a bleeding rate of 12.9% at 12 weeks [36]. More recently, a prospective cohort study of 188 patients with upper extremity DVT treated with DOACs (54% rivaroxaban; 30% apixaban; 10% edoxaban; 6% dabigatran), including 29% with active cancer and 33% with catheter-related or pacemaker-related DVTs, reported more reassuring findings, although the results are not specific to patients with cancer [37]. During treatment with DOACs, recurrent VTE occurred in 0.9 per 100 patient-years, major bleeding in 1.7 per 100 patient-years and all-cause deaths in 6.0 per 100 patient-years [37]. Based on the available evidence and expert opinion, the consensus group recommends that choice of anticoagulant for the treatment of upper extremity and catheter-related VTE be individualized similarly as for proximal lower limb DVT and PE based on the factors discussed in this paper.

### 4.4. Risk of Bleeding

The risk of major bleeding was higher with DOACs than LMWH in both the Hokusai-VTE Cancer and SELECT-D trials, although rates of major bleeding were similar in the CARAVAGGIO, ADAM-VTE, and CASTA-DIVA trials (Table 1) [16,17,18,27,28]. Overall, pooled estimates from meta-analyses have reported a non-significantly higher rate of major bleeding complications among patients with CAT receiving a DOAC as compared to LMWH (HR:1.26; 95% CI 0.84–1.90 and RR: 1.36; 95% CI 0.55 to 3.35) [28,38]. Hence, identifying patients at higher risk of bleeding complications might be helpful to tailor anticoagulation in this patient population. In the Hokusai-VTE Cancer trial, the excess bleeding risk was attributable mainly to patients with GI cancer, of whom 12.7% (21/165) in the edoxaban arm experienced major bleeding as compared to 3.6% (5/140) in the dalteparin arm [39]. Additionally, for most of the edoxaban-treated patients with major bleeding, the site of the bleed was the upper GI tract (16 of 21 cases), with the remaining sites being the lower GI tract, epistaxis, and retro-peritoneum. By contrast, only one of the five cases of major bleeding in the dalteparin-treated patients was at a GI site [39]. Similarly, in SELECT-D, 45.5% (5/11) of all major bleeding episodes in rivaroxaban-treated patients occurred in the GI tract [17]. Like Hokusai-VTE Cancer, the SELECT-D trial also showed a signal for a higher risk of bleeding in patients with GI cancer, with the data safety monitoring committee of the SELECT-D trial noting a non-significant increase in major bleeding events in 19 patients with esophageal or gastroesophageal junction cancers after a safety review of the first 220 patients [17]. Patients with those cancers were subsequently excluded from enrolment. The CARAVAGGIO trial did not report any difference in major bleeding complications between patients receiving apixaban or dalteparin [18,40]. A total of 1.9% (11/576) and 1.7% (10/579) of patients had GI major bleeding complications among those receiving apixaban and dalteparin, respectively [40]. The reasons for the discrepancy in GI major bleeding are unclear and may be related to differences in baseline characteristics (tumor types, etc.) among the included patients in the different studies or related to the properties of the individual DOACs (once vs. twice a day, topical mucosal anticoagulant effect, etc.). A recent observational study reported that apixaban had a higher rate of major bleeding complications in patients with luminal GI cancers compared to those with non-GI cancers (15.6 vs. 3.7 per 100 person-years, *p* = 0.004) and compared to enoxaparin in patients with luminal GI cancer (15.6 vs. 3.2, *p* = 0.04) [41]. Hence, all DOACs should be use cautiously in patients with GI cancers, especially in those with unresected luminal tumors.

Other patient characteristics are also important for clinicians to consider. Subgroup analyses of major bleeding in the Hokusai-VTE Cancer safety population suggest that, in addition to GI cancer, other features associated with a higher risk of major bleeding include urothelial cancer, renal impairment, thrombocytopenia, intracranial malignancy, regionally advanced or metastatic cancer, recent surgery, and use of antiplatelet agents or bevacizumab [16]. Analysis of clinically relevant bleeding events in the CATCH trial confirmed that intracranial malignancy increases the risk of bleeding regardless of the type of anticoagulation [42]. Age > 75 years was also significantly associated with an increased risk of clinically relevant bleeding in this analysis [42].

The Hokusai-VTE Cancer, SELECT-D, and CARAVAGGIO trials have reported greater proportions of DOAC-treated patients who experienced clinically relevant non-major bleeding (CRNMB) events with HRs of 1.38 (95% CI 0.98 to 1.94), 3.76 (95% CI 1.63 to 8.69), and 1.42 (95% CI 0.88 to 2.30) for edoxaban, rivaroxaban, and apixaban, respectively [16,17,18]. In the Hokusai-VTE Cancer trial, CRNMB events were numerically more common for GI, epistaxis, hematuria, or abnormal uterine bleeding in patients receiving edoxaban compared to those receiving dalteparin [39]. In SELECT-D, the significantly higher rates of CRNMB in patients receiving rivaroxaban were due to GI and GU bleeding, which accounted for 9 and 11 of the 25 CRNMB events, respectively [17]. In CARAVAGGIO, the numerical increase in CRNMB was largely due to bleeding in the GU and upper airway tracts, which accounted for 20 and 14 cases, respectively, of the 59 CRNMB events in patients receiving apixaban [40]. Additionally, patients with GI cancer appeared to be at higher risk of bleeding events with DOAC, with 13.2% (19/144) of patients with GI cancer who were treated with apixaban experiencing CRNMB as compared to 4.9% (7/144) in the dalteparin arm [40]. Overall, GI or GU CRNMB may be more common in patients receiving a DOAC than in those treated with LMWH.

#### 4.4.1. Features Consistent with a High Risk of GI Bleeding

Given that bleeding rates appear to be higher with DOACs in patients with GI tumors or those on treatments such as bevacizumab that are associated with tumor necrosis and bleeding [16,17,39,40], the committee suggests considering the use of LMWH for patients with these or other features that are associated with a high risk of GI bleeding, such as angiodysplasia, GI lesion, previous variceal bleed, or treatment-associated mucosal toxicity. The risk of GI perforation and/or hemorrhage associated with a patient’s anticancer therapies should be taken into consideration regardless of which anticoagulant is selected.

#### 4.4.2. Thrombocytopenia

Thrombocytopenia increases the risk of bleeding complications in patients with CAT [43]. Unfortunately, there is limited evidence to guide management in patients with platelet counts <50,000 platelets/mL. The CLOT trial excluded patients with baseline platelet counts <75,000 platelets/mL, while the Hokusai-VTE Cancer, CARAVAGGIO, and SELECT-D trials excluded patients with baseline platelet counts of less than 50,000, 75,000, and 100,000 platelets/mL, respectively [8,16,17,18]. Guidance from the SSC of the ISTH suggests that therapeutic dose of anticoagulation can be used for patients with platelet count of ≥50,000 platelets/mL [44]. In patients with platelet counts of less than 50,000 platelets/mL, 50% or prophylactic dose LMWH may be used or full-dose anticoagulation with platelet transfusion support may be considered [44]. The Canadian consensus committee suggests that LMWH is preferred in these patients but recommends seeking an expert opinion from a specialized physician when initiating anticoagulation in the setting of severe thrombocytopenia (i.e., platelet counts <50,000/mL). In cases of transient thrombocytopenia due to anticancer therapies, clinical judgment should be used to determine whether the anticoagulant needs to be dose-reduced or temporarily held until platelet levels recover to ≥50,000 platelets/mL.

#### 4.4.3. Intracranial Lesions

As there were few patients with intracranial tumors (primary brain tumor or brain metastasis) included in the DOAC trials (none in CARAVAGGIO, 7% of patients (74/1046) in Hokusai VTE Cancer, and only 1% of patients in SELECT-D), there are limited data regarding the safety of this anticoagulant class in these patients [16,17,18]. Some reassurance may be provided by a retrospective cohort study of the cumulative incidence of intracranial hemorrhage (ICH) with DOAC vs. LMWH in patients with brain tumors and VTE [45]. In this study, no ICH was noted among 20 patients with primary brain tumors treated with DOACs, while the cumulative incidence among the 47 patients treated with LMWH was 37%. Among 105 patients with brain metastases, the cumulative incidence of ICH was 11% among those treated with DOAC and 18% in those treated with LMWH [45]. Similarly, an international two-center study suggested comparable safety of LMWH and DOACs in patients with brain metastases. The 12-month cumulative incidence of major ICH was 5.1% in DOAC-treated patients and 11.1% in those treated with LMWH (HR: 0.45; 95% CI 0.09 to 2.21) [46]. When anticoagulation was analyzed as a time-varying covariate, the risk of any ICH did not differ between DOAC- and LMWH-treated patients (HR: 0.98; 95% CI 0.28 to 3.40) [46]. Finally, a single-center retrospective chart review of 125 patients with primary and metastatic brain tumors on anticoagulation reported rates of major bleeding of 26% and 9.6% in patients receiving LMWH or DOAC, respectively [47]. Patients receiving DOAC also had a lower rate of ICH compared to those receiving LMWH (5.8% vs. 15%) [47]. Nevertheless, given the small numbers and the limitations of retrospective studies, as well the shorter half-life of LMWH, the consensus committee suggests considering the initial use of LMWH for patients with CAT and high-risk intracranial lesions (e.g., glioma).

#### 4.4.4. Hepatic and Renal Impairment

Patients with functional hepatic impairment may have reduced ability to metabolize DOACs, all of which are at least partially metabolized by cytochrome P450 (CYP) enzymes [48,49,50]. Patients with significant liver disease are thus considered to be at higher risk of bleeding when treated with DOACs and were excluded from clinical trials. For these reasons, none of the DOACs are recommended for use in patients meeting criteria for Child-Pugh class C [48,49,50]. Rivaroxaban is contraindicated in patients with hepatic disease (including Child-Pugh class B and C) associated with coagulopathy and having clinically relevant bleeding risk [48]. Apixaban should be used with caution in patients with mild or moderate hepatic impairment (Child-Pugh class A or B) [49]. With edoxaban, patients with Child-Pugh class A or B exhibited comparable pharmacokinetics and pharmacodynamics to healthy controls [50].

The previous iteration of the Canadian expert consensus treatment algorithm suggested that LMWH might be preferable to DOAC in patients with CAT and a creatinine clearance of 30–50 mL/min, especially if additional risk factors for bleeding were present [19]. This recommendation was based on the limited evidence available at the time suggesting a potentially elevated risk of bleeding with edoxaban in these patients [16,19]. However, this recommendation was not supported by the CARAVAGGIO trial, which found no significant between-treatment differences in rates of major bleeding in patients with creatinine clearance of 30–80 mL/min treated with apixaban or LMWH [18]. Thus, the consensus committee currently recommends that clinicians follow product monograph recommendations for contraindications and dose adjustment of anticoagulants in patients with impaired renal function (Table 2).

#### 4.4.5. Use of Antiplatelet Agents

The use of either dual antiplatelet therapy or higher doses of acetylsalicylic acid (ASA) was not permitted in the DOAC vs. LMWH RCTs, with concomitant ASA at doses >75 mg, >100 mg, and >165 mg daily being exclusion criteria in the SELECT-D, Hokusai-VTE Cancer, and CARAVAGGIO trials, respectively [16,17,18]. However, even at low doses, ASA is known to increase the risk of upper GI bleeds, a risk which appears to be increased when it is used in conjunction with oral anticoagulants [54,55]. This was confirmed by subgroup analysis of data from the Hokusai-VTE cancer trial, which showed a numerical increase in the risk of major bleeding in DOAC-treated patients on concomitant antiplatelet therapy [16]. Similarly, in the CARAVAGGIO trial, 22.7% (5/22) of patients on concomitant antiplatelet therapy treated with apixaban experienced major bleeding as compared to 11.8% (68/576) of apixaban-treated patients without antiplatelet therapy [40]. No major bleeding events were reported among the 23 patients in the LMWH arm on concomitant antiplatelet therapy, while 12.8% (74/579) of those without antiplatelet therapy had a major bleed [40]. Given this, the consensus committee recommends that the indication for antiplatelet agents be reassessed, and discontinuation should be considered in the absence of a strong indication in patients with new diagnosis of CAT. Shared decision-making with other health care providers would be warranted in these circumstances.

### 4.5. Drug–Drug Interactions

Polypharmacy is common in patients with cancer, who are often treated with multiple anticancer and supportive therapies. It is thus important to evaluate the potential for drug–drug interactions when selecting the appropriate anticoagulant therapy for CAT. All DOACs are substrates of P-glycoprotein, and apixaban and rivaroxaban are also substrates of CYP3A4, so therapies that affect P-glycoprotein or CYP3A4 metabolism have the potential to interact with DOACs [56]. Numerous anticancer therapies are inhibitors or inducers of the P-glycoprotein and/or CYP3A4 pathways, with the potential to interact with DOACs [57]. Anticancer therapies for which the potential for drug–drug interactions with DOACs should be considered include abiraterone, acalabrutinib, afatinib, ceritinib, cyclosporine, cobimetinib, crizotinib, dabrafenib, dasatinib, dexamethasone, doxorubicin, enzalutamide, erdafitinib, ibrutinib, idelalisib, imatinib, ipilimumab, lapatinib, mitotane, neratinib, nilotinib, nintedanib, niraparib, olaparib, panobinostat, ponatinib, ribociclib, sunitinib, tacrolimus, tamoxifen, trametinib, trastuzumab emtansine, vandetanib, vemurafenib, and vinblastine [57].

However, assessing the potential for clinically significant interactions is complex as not all potential interactions appear to be clinically important [58]. In fact, sub-analysis of patients treated concomitantly with anticancer agents and anticoagulants in the CARAVAGGIO trial found no significant differences in rates of major bleeding, recurrent VTE, or death between the DOAC and LMWH arms [59]. Table 3 lists drug–drug interactions with DOACs that have been shown to have clinical relevance. Notably, a recent registry of the ISTH including 202 patients receiving concurrent DOACs and targeted anticancer therapies has reported a high rate of bleeding complications in patients receiving Bruton’s tyrosine kinase (BTK) inhibitors [60]. A recent observational study has also reported a higher risk of bleeding in patients receiving concurrent vascular endothelial growth factor receptor (VEGFR) tyrosine kinase inhibitors (TKIs) and LMWH [61]. The study sample size was inadequate for between-treatment comparisons with concurrent TKIs and DOACs. Given the complexity of the therapeutic regimens used to treat many patients with cancer, the consensus committee recommends that patients with CAT be referred for a pharmacist-led drug interaction evaluation, which should be repeated if cancer management changes. Alternatively, online drug–drug interaction applications or websites can be helpful, although previous publications have highlighted important differences in the accuracy and quality of these tools [62,63]. However, when using such tools, clinicians must keep in mind that the majority of reported interactions are theoretical rather than having been proven to be associated with decreased drug levels (and thus thrombosis) or increased drug levels (and thus bleeding).

### 4.6. Other Factors to Consider

#### 4.6.1. Patient and Clinician Preference and Drug Cost

In addition to efficacy, safety, and potential for drug–drug interactions, clinicians and patients should together consider drug cost and accessibility, taking into account the patient’s individual situation, as well as publicly or privately funded drug plan coverage. Shared decision-making may help improve adherence to therapy, and, in turn, lead to improved anticoagulant effectiveness.

#### 4.6.2. Body Weight

There is a paucity of evidence regarding the use of DOACs or LMWHs in patients with cancer at the extremes of body weight. In the Hokusai-VTE Cancer and CARAVAGGIO trials, neither low (≤60 kg) nor high (>90 kg) body weight had a significant impact on the risk of recurrent VTE or major bleeding, although there were few patients in these extreme weight categories who were included in the trials [16,18]. It should be noted that the daily dose of edoxaban was reduced in the Hokusai-VTE Cancer trial to 30 mg from 60 mg in patients with a body weight ≤60 kg [16]. Dose adjustment based on body weight is not recommended in the product monographs for apixaban or rivaroxaban [48,49].

Per the approved indication, the dose of the LMWH dalteparin was capped at 18,000 IU daily in the DOAC trials in CAT [16,17,18]. However, prior non-cancer studies have shown that body weight does not have an important effect on anti-Xa activity levels achieved with weight-based doses of enoxaparin, dalteparin, and tinzaparin for patients weighing up to 144 kg, 190 kg, and 165 kg, respectively [64,65,66]. Furthermore, a meta-analysis of data including 921 patients with a body mass index (BMI) of 30 kg/m^2^ or more showed no increased risk of bleeding compared with non-obese patients (BMI < 30 kg/m^2^) who received weight-adjusted, “uncapped” LMWH [67]. Therapeutic weight-adjusted dosing, without capping, is therefore suggested for LMWH use [68].

Based on meta-analyses and phase 4 studies, the SCC of the ISTH suggests that standard doses of rivaroxaban or apixaban are among appropriate anticoagulant options for the treatment and prevention of VTE in the general, non-cancer population, regardless of BMI and weight [69]. Fewer supportive data exist for apixaban than rivaroxaban. VKA and weight based LMWH are also considered options. The SCC suggests not to use dabigatran or edoxaban in patients with BMI > 40 kg/m^2^ or weight > 120 kg, given unconvincing data for dabigatran, and lack of clinical or pharmacokinetic and pharmacodynamic data for edoxaban [69]. These suggestions are not specifically for CAT but for the overall management of VTE. Based on the limited evidence base, the consensus committee recommends that LMWH be considered in patients with weight > 150 kg and an agent with weight-adjustable dosing, such as edoxaban or LMWH, be considered in patients with weight < 50 kg.

#### 4.6.3. Burden of Cancer and Burden of VTE

Limited data are available regarding the effects of the burden of cancer and burden of VTE on the efficacy and safety of anticoagulation. Cross-study comparison is limited by differences between clinical trials in baseline characteristics reflecting cancer burden (Table 4), as well as in overall mortality rates (Table 1), which suggest the overall burden of cancer may have been higher in the LMWH vs. VKA trials than in the DOAC vs. LMWH trials [8,9,16,17,18,24,25,26,27,28]. It is also important to note that patients who required thrombolysis or who underwent inferior vena cava filter insertion were excluded from the trials. In the absence of RCT data, the consensus committee recommends that initial therapy with LMWH be considered for patients with severe symptoms of thrombosis, including patients with iliofemoral DVT, extensive PE, or sub-massive PE, and any patients who received thrombolysis.

#### 4.6.4. Abnormal Uterine Bleeding

Abnormal uterine bleeding is a common but under-reported complication of anticoagulation that is thought to occur in up to 70% of women of reproductive age receiving anticoagulation [70]. The risk of anticoagulant-related abnormal uterine bleeding varies by DOAC, with apixaban and edoxaban having statistically similar relative risks to VKA and rivaroxaban appearing to double the risk as compared to VKA (RR: 2.10; *p* < 0.01) [70,71]. Estimates of the risk of abnormal uterine bleeding with LMWH monotherapy are not available. However, despite the lack of data for LMWH, for women who experience abnormal uterine bleeding while on DOAC or with a history of abnormal uterine bleeding associated with a DOAC, the consensus committee recommends consideration of LMWH. Other management options for abnormal uterine bleeding secondary to anticoagulation include tranexamic acid, and hormonal therapy, such as combined oral contraceptives, the levonorgestrel intrauterine device, and depo-medroxyprogesterone acetate, although estrogen-based regimens should be avoided in patients who are no longer on anticoagulation [70]. Adjunctive iron therapy to manage iron deficiency anemia, as well as consultation with gynecology may also be considered.

#### 4.6.5. Significant GI Surgery or Absorption Disorders

DOACs are absorbed by different sites throughout the GI tract, with edoxaban being primarily absorbed by the proximal small intestine, rivaroxaban absorbed by both the stomach and proximal intestine and apixaban absorbed throughout the GI tract including significant (>50%) absorption in the distal small bowel or ascending colon [72]. Given the GI absorption of DOACs, there is concern regarding their use in patients who, because of GI surgery or other disorders, have a significant reduction in intestinal absorptive surface. However, there is limited evidence regarding the pharmacodynamics or clinical outcomes associated with DOACs in these patients [72]. The SSC of the ISTH recommends not using DOACs for treatment or prevention of VTE in the acute setting after bariatric surgery due to concerns of decreased gastric absorption [69]. Such a recommendation might apply to cancer patients who have undergone a Whipple’s procedure. Initiation of anticoagulation with LMWH or another parenteral agent is recommended in such cases. Until a greater evidence base is available, it is reasonable to consider LMWH for patients with impaired GI absorption.

### 4.7. Reassessing Treatment for Secondary Prophylaxis

Prospective studies that evaluated anticoagulation therapy beyond 6 months include the DALTECAN, TICAT, Hokusai-VTE Cancer, and SELECT-D studies [16,73,74,75]. In the DALTECAN prospective cohort study, 55% of the 334 patients with VTE and active cancer who were treated with dalteparin completed 6 months of therapy, and 33% completed 12 months [73]. Therapy beyond 6 months was not associated with an increased risk of major bleeding or recurrent VTE as compared to the first 6 months. Similarly, the TICAT study, which evaluated the safety of long-term tinzaparin in 247 patients with CAT, reported no significant difference in recurrent VTE or clinically relevant bleeding for months 1 to 6 compared with months 7 to 12 [74]. The Hokusai-VTE Cancer trial demonstrated acceptable efficacy and safety profiles during the 12-month treatment period [16]. However, the median therapy duration was approximately 6 months, and rates of recurrent VTE and major bleeding beyond the initial 6 months were not reported. After 6 months of treatment in the SELECT-D trial, patients with active cancer and residual DVT or index PE were eligible for re-randomization to rivaroxaban or placebo [75]. Of the 92 patients who were re-randomized, 4% of those treated with rivaroxaban experienced recurrent VTE as compared to 14% of those treated with placebo (HR: 0.32; 95% CI 0.06 to 1.58), while major bleeding occurred in 0% of patients treated with placebo and 5% of those randomized to rivaroxaban [75]. Although there are no prospective data assessing the efficacy and safety of anticoagulation for secondary prevention of recurrent VTE beyond 12 months, retrospective cohort studies have shown that the risk of recurrent VTE and major bleeding remains elevated beyond 12 months in this patient population [76,77].

Several studies have also investigated the safety and efficacy of prophylactic doses of DOACs for the extended treatment of VTE [78,79]. However, few patients with active cancer were included in these trials. and it is unclear whether the results can be generalized to this population. The ongoing API-CAT study, which aims to determine whether a low-dose regimen of apixaban is non inferior to a full-dose regimen of apixaban for the prevention of recurrent VTE in patients with active cancer who have completed at least 6 months of anticoagulant therapy for treating VTE, may help clarify this issue [80].

Considering the weak evidence supporting long-term secondary prophylaxis in patients with CAT, most guidelines suggest that the decision to continue anticoagulation beyond the initial 6 months should be individualized [19,20,21,22,23]. Most of the studies supporting long-term secondary prevention in patients with cancer assessed therapeutic dosing of anticoagulation (LMWH or DOAC). However, decision on duration and dosing of anticoagulation for long-term secondary prevention should be made based on the patient’s risk factors for VTE recurrence and bleeding complications and re-assessed on a regular basis. The consensus committee suggests that patients’ risk factors for VTE recurrence and bleeding should be reassessed regularly (e.g., at least every 3 months or sooner if there are changes in cancer status or management).

## 5. Conclusions

Selection of anticoagulation for the treatment of CAT should be individualized based on the patient’s bleeding risk, type of cancer, and potential for drug–drug interactions, as well as patient and clinician preferences. Anticoagulant therapy should be reassessed on a regular basis as the patient’s cancer status and management change over time.

## Figures and Tables

**Table 1:** Randomized controlled trials for the acute treatment of cancer-associated thrombosis.

Reference (Study Name)	Patients (*n*)	Intervention	Duration (Months)	Major Bleeding (%) ^b^	Recurrent VTE (%) ^b^	Death (%) ^b^
LMWH compared with VKA						
Meyer et al. 2002 (CANTHANOX) [24]	67	Enoxaparin 1.5 mg/kg daily	3	7	3	22.7
	71	VKA		16	4.2	11.3
Lee et al., 2003 (CLOT) [8]	336	Dalteparin 200 IU/kg daily for 1 month, and then 150 IU/kg	6	4	9	39
	336	VKA		6	17	41
Deitcher et al. 2006 (ONCENOX) ^a^ [25]	29	Enoxaparin 1 mg/kg daily	3	6.5	6.9	6.5
	32	Enoxaparin 1.5 mg/kg daily		11.1	6.3	19.4
	30	VKA		2.9	10	8.8
Hull et al. 2006 (LITE) [26]	100	Tinzaparin 175 IU/kg daily	3	7	6	19
	100	VKA		7	10	20
Lee et al. 2015 (CATCH) [9]	449	Tinzaparin 175 IU/kg daily	6	2.7	7.2	33
	451	VKA		2.4	10.5	31
DOAC compared with LMWH						
Raskob et al. 2018 (Hokusai-VTE Cancer) [16]	522	LMWH for ≥5 days, and then edoxaban 60 mg daily	12	6.9	7.9	39.5
	524	Dalteparin 200 IU/kg daily for 1 month, and then 150 IU/kg		4.0	11.3	36.6
Young et al. 2018 (SELECT-D) [17]	203	Rivaroxaban 15 mg twice daily for 3 weeks, and then 20 mg daily	6	6	4	25
	203	Dalteparin 200 IU/kg daily for 1 month, and then 150 IU/kg		4	11	30
McBane et al. 2020 (ADAM-VTE) [27]	145	Apixaban 10 mg twice daily for 7 days, and then 5 mg twice daily	6	0	0.7	16
	142	Dalteparin 200 IU/kg daily for 1 month, and then 150 IU/kg		1.4	6.3	11
Agnelli et al. 2020 (CARAVAGGIO) [18]	576	Apixaban 10 mg twice daily for 7 days, and then 5 mg twice daily	6	3.8	5.6	23.4
	579	Dalteparin 200 IU/kg daily for 1 month, and then 150 IU/kg		4.0	7.9	26.4
Planquette et al. 2021 (CASTA-DIVA) [28]	74	Rivaroxaban 15 mg twice daily for 3 weeks, and then 20 mg daily	3	1.4	6.0	25.7
	84	Dalteparin 200 IU/kg daily for 1 month, and then 150 IU/kg		3.7	9.5	23.8

^a^ All groups started with enoxaparin 1 mg/kg twice daily for 5 days. ^b^ Number of events divided by the number of patients included in each arm. DOAC = direct-acting oral anticoagulant; LMWH = low-molecular-weight heparin; VKA = vitamin K antagonist; VTE = venous thromboembolism.

**Table 2:** Product monograph dosing recommendations according to creatinine clearance.

Anticoagulant	Creatinine Clearance (mL/min)			
	<15 or Dialysis	15–29	30–50	>50
LMWH				
Dalteparin [51]	Dose reduction should be considered ^a^	Dose reduction should be considered ^a^	200 IU/kg once daily for 1 month, and then 150 IU/kg	200 IU/kg once daily for 1 month, and then 150 IU/kg
Enoxaparin [52]	100 IU/kg once daily	100 IU/kg once daily	100 IU/kg twice daily	100 IU/kg twice daily
Tinzaparin [53]	175 IU/kg once daily ^a^	175 IU/kg once daily ^a^	175 IU/kg once daily	175 IU/kg once daily
DOAC				
Apixaban [49]	Not recommended	10 mg twice daily for 7 days, and then 5 mg twice daily ^b^	10 mg twice daily for 7 days, and then 5 mg twice daily ^b^	10 mg twice daily for 7 days, and then 5 mg twice daily ^b^
Edoxaban [50]	Not recommended	Not recommended	30 mg once daily (following initial 5–10 days of LMWH)	60 mg once daily (following initial 5–10 days of LMWH)
Rivaroxaban [48]	Not recommended	15 mg twice daily for 3 weeks, and then 20 mg once daily ^b^	15 mg twice daily for 3 weeks, and then 20 mg once daily ^b^	15 mg twice daily for 3 weeks, and then 20 mg once daily ^b^

^a^ Use with caution when treating patients with creatinine clearance <30 mL/min; see product monograph for dosing in hemodialysis and hemofiltration. ^b^ Must be used with caution in patients with creatinine clearance 15–29 mL/min due to potentially higher bleeding risks. DOAC = direct-acting oral anticoagulant; LMWH = low-molecular-weight heparin.

**Table 3:** Clinically significant drug-drug interactions with direct-acting oral anticoagulants [58,60].

Interacting Drug	Outcome	Proposed Mechanism of Interaction
Acalabrutinib	↑ bleeding risk	Weak CYP3A4 inhibitor/antiplatelet effect
Amiodarone	↑ bleeding risk	Weak CYP3A4/P-gp inhibitor
Carbamazepine	↓ antithrombotic efficacy	Strong CYP3A4/P-gp inducer
Clarithromycin	↑ bleeding risk	Strong CYP3A4/P-gp inhibitor
Cyclosporine	↑ bleeding risk	Weak CYP3A4/P-gp inhibitor
Diltiazem	↑ bleeding risk	Moderate CYP3A4/P-gp inhibitor
Efavirenz	↓ antithrombotic efficacy	Moderate CYP3A4 inducer
Fluconazole	↑ bleeding risk	Moderate CYP3A4 inhibitor
Ibrutinib	↑ bleeding risk	Weak CYP3A4/P-gp inhibitor/antiplatelet effect
Loperamide	↑ bleeding risk	Mechanism unclear
Miconazole (topical)	↑ bleeding risk	Mechanism unclear
Nevirapine	↓ antithrombotic efficacy	Weak CYP3A4 inducer
Oxcarbazepine	↓ antithrombotic efficacy	Weak CYP3A4 inducer
Phenobarbital	↓ antithrombotic efficacy	Strong CYP3A4 inducer
Phenytoin	↓ antithrombotic efficacy	Strong CYP3A4/P-gp inducer
Quinidine	↑ bleeding risk	Moderate P-gp inhibitor
Rifampicin	↓ antithrombotic efficacy	Strong CYP3A4/P-gp inducer
Ritonavir	↑ bleeding risk	Strong CYP3A4/P-gp inhibitor
Tocilizumab	↓ antithrombotic efficacy	Indirect P-gp inducer
Verapamil	↑ bleeding risk	Moderate CYP3A4/P-gp inhibitor

CYP3A4 = cytochrome P450 3A4; P-gp = P-glycoprotein.

**Table 4:** Baseline characteristics of selected randomized controlled trials for the acute treatment of cancer-associated thrombosis.

Reference (Study Name)	Anticoagulant	Age (Years)	Metastatic Cancer (%)	Cancer Therapy (%)	ECOG PS 2 (%)	Top 3 Cancer Types
Lee et al., 2003 (CLOT) [8]	Dalteparin	62	66	79	35	Breast Colorectal Lung
	VKA	63	69	77	36	
Lee et al., 2015 (CATCH) [9]	Tinzaparin	60	66	51	24	Gynecologic Lung Upper GI
	VKA	59	63	55	23	
Raskob et al., 2018 (Hokusai-VTE Cancer) [16]	Edoxaban	64	52	72	24	Colorectal Lung Genitourinary
	Dalteparin	64	53	63	24	
Young et al., 2018 (SELECT-D) [17]	Rivaroxaban	67	58	69	26	Colorectal Lung Breast
	Dalteparin	67	58	70	21	
McBane et al., 2020 (ADAM-VTE) [27]	Apixaban	64	65	73	13	Colorectal Lung Pancreatic
	Dalteparin	64	66	74	8	
Agnelli et al., 2020 (CARAVAGGIO) [18]	Apixaban	67	68 ^a^	61	19	Colorectal Lung Breast
	Dalteparin	67	68 ^a^	63	23	
Planquette et al., 2021 (CASTA-DIVA) [28]	Rivaroxaban	69	77	70	NR	Colorectal Lung Breast
	Dalteparin	71	76	74	NR	

^a^ Combination of locally advanced and metastatic disease. DOAC = direct-acting oral anticoagulant; ECOG = Eastern Cooperative Oncology Group; PS = performance status; VKA = vitamin K antagonist.

## Appendix A

Abstracts in the PubMed database from January 2018 to March 4, 2021 were searched used the following search string: (“Neoplasms”[Mesh] OR “Carcinoma”[Mesh]) AND (“Venous Thromboembolism”[Mesh] OR “Pulmonary Embolism”[Mesh] OR “Venous Thrombosis”[Mesh]) AND (“Anticoagulants”[Mesh] OR “Heparin, Low-Molecular-Weight”[Mesh] OR “Warfarin”[Mesh] OR “apixaban”[Supplementary Concept] OR “Dabigatran”[Mesh] OR “edoxaban”[Supplementary Concept] OR “Rivaroxaban”[Mesh]). The results were restricted to English-language publications focusing on humans.

Abstracts presented at the 2020 American Society of Hematology Annual Meeting were searched using the following terms: (cancer) AND (thromboembolism OR thrombosis OR embolism) AND (anticoagulant OR LMWH OR warfarin OR apixaban OR dabigatran OR edoxaban OR dalteparin OR enoxaparin OR tinzaparin).
